# Impact of routine percutaneous coronary intervention after out-of-hospital cardiac arrest due to ventricular fibrillation

**DOI:** 10.1186/cc10227

**Published:** 2011-05-11

**Authors:** Pierrick Cronier, Philippe Vignon, Koceila Bouferrache, Philippe Aegerter, Cyril Charron, François Templier, Samuel Castro, Rami El Mahmoud, Cécile Lory, Nicolas Pichon, Olivier Dubourg, Antoine Vieillard-Baron

**Affiliations:** 1 Cardiological Department, Section Thorax - Vascular diseases - Abdomen - Metabolism, University Hospital Ambroise Paré, 9 avenue Charles de Gaulle, 92104 Boulogne, France; 2 Faculté de Médecine Paris Ile de France Ouest, Université de Versailles Saint Quentin en Yvelines, 78000 Versailles, France; 3 Intensive Care Unit, CHU de Limoges, 2 avenue Martin Luther King, 87042 Limoges, France; Center of Clinical Investigation, INSERM 0801, 87042 Limoges, France; University of Limoges, 87000 Limoges, France; 4 Intensive Care Unit, Section Thorax - Vascular diseases - Abdomen - Metabolism, University Hospital Ambroise Paré, 9 avenue Charles de Gaulle, 92104 Boulogne, France; 5 Department of Biostatistics and Clinical Research Unit, University Hospital Ambroise Paré, 9 avenue Charles de Gaulle, 92104 Boulogne, France; 6 SAMU 92, University Hospital Raymond Poincaré, 104 Boulevard Raymond Poincaré, 92000 Garches, France

## Abstract

**Introduction:**

Since 2003, we have routinely used percutaneous coronary intervention (PCI) and mild therapeutic hypothermia (MTH) to treat patients < 80 years of age after out-of-hospital cardiac arrest (OHCA) related to ventricular fibrillation. The aim of our study was to evaluate the prognostic impact of routine PCI in association with MTH and the potential influence of age.

**Methods:**

We studied 111 consecutive patients resuscitated successfully following OHCA related to shock-sensitive rhythm. They were divided into five groups according to age: < 45 years (*n *= 22, group 1), 45 to 54 years (*n *= 27, group 2), 55 to 64 years (*n *= 22, group 3), 65 to 74 years (*n *= 23, group 4) and ≥75 years (*n *= 17, group 5). Emergency coronary angiography was performed in hemodynamically stable patients < 80 years old, regardless of the electrocardiogram pattern. MTH was targeted to a core temperature of 32°C to 34°C for 24 hours.

**Results:**

Most patients (73%) had coronary heart disease, although its incidence in group 1 was lower than in other groups (41% versus 81%; *P *= 0.01). In group 1, all patients but one underwent coronary angiography, and 33% of them underwent associated PCI. In group 5, only 53% of patients underwent a coronary angiography and 44% underwent PCI. Overall in-hospital survival was 54%, ranging between 52% and 64% in groups 1 to 4 and 24% in group 5. Time from collapse to return of spontaneous circulation was associated with mortality (odds ratio (OR) = 1.05 (25th to 75th percentile range, 1.03 to 1.08); *P *< 0.001), whereas PCI was associated with survival (OR = 0.30 (25th to 75th percentile range, 0.11 to 0.79); *P *= 0.01).

**Conclusions:**

We suggest that routine coronary angiography with potentially associated PCI may favorably alter the prognosis of resuscitated patients with stable hemodynamics who are treated with MTH after OHCA related to ventricular fibrillation. Although age was not an independent cause of death, the clinical relevance of this therapeutic strategy remains to be determined in older people.

## Introduction

Coronary artery disease is the most common cause of sudden cardiac death, and ventricular fibrillation (VF) due to ischemic cardiomyopathy is responsible for more than 50% of out-of-hospital cardiac arrest (OHCA) cases [1]. In this setting, coronary angiography associated with percutaneous coronary intervention (PCI) has been recommended in the presence of ST elevation [2], since it may decrease post-cardiac arrest syndrome and improve survival [3].

Recently, mild therapeutic hypothermia (MTH) has been reported to improve neurological outcomes in patients who have sustained OHCA caused by VF [4,5]. Some studies have suggested that coronary angiography associated with MTH treatment in patients with OHCA and ST-segment elevation may improve survival [6]. Sunde *et al*. [7] also reported promising results in terms of prognosis when applying a standardized treatment including PCI and MTH in these patients. Accordingly, PCI and MTH are currently recommended by the American guidelines for cardiopulmonary resuscitation in adult patients under 75 years old who have sustained OHCA secondary to VF with ST elevation [2]. However, ST elevation is known to be a poor predictor of acute coronary occlusion after cardiac arrest [8]. In addition, the prognostic impact of PCI remains debatable [9,10], and most trials evaluating the potential impact of MTH and emergency PCI on survival have been performed in patients under 75 years of age. The few studies which have assessed prognostic factors in the older adult patient population have been conducted prior to the routine use of PCI and MTH and have provided conflicting results [11,12].

The aim of our study was to evaluate the prognostic impact of routine coronary angiography, with PCI if necessary and regardless of electrocardiogram (ECG) pattern, in patients treated with MTH for resuscitated OHCA related to VF and to assess the potential influence of age.

## Materials and methods

### Study design

This prospective study was conducted from January 2003 to September 2008 in the intensive care units (ICUs) of two university hospitals. The study population consisted of all consecutive patients resuscitated successfully following OHCA related to shock-sensitive rhythm. The criteria for inclusion were cardiac arrest with ventricular arrhythmia (that is, requiring electric shock therapy) regardless of cause, as well as the need for mechanical ventilation. Exclusion criteria were age < 18 years or the absence of information regarding the time from collapse to return of spontaneous circulation (ROSC). Since systematic coronary angiography and MTH have been performed routinely in all patients under 80 years of age in the two participating ICUs since January 2003 as a standard of care, our study was considered part of routine clinical practice and thus no informed consent was required from the patients' next of kin by the Ethics Committee of the Ambroise Paré Hospital.

### Protocol of care

Before their admission to the ICU, emergency coronary angiography was performed in hemodynamically stable patients under 80 years old, regardless of the ECG pattern. Hemodynamic stability was defined as systolic arterial pressure > 90 mmHg with or without epinephrine infusion for at least 30 minutes. The decision to perform PCI was based on the identification of either an occluded coronary vessel or significant stenosis of the coronary artery supplying the myocardial territory suspected of acute ischemia. In patients with unstable hemodynamics, coronary angiography was not performed.

MTH was initiated in the angiography room and maintained in the ICU using an initial infusion of 4°C saline (500 mL to 1 L) and either external cooling or a specific intravascular catheter connected to a cooling system (CoolGard; Zoll Medical Corp, Chelmsford, MA, USA) to lower and stabilize the patient's core temperature to 32°C to 34°C as recommended [2]. The target temperature was maintained for 24 hours with a progressive normalization of body temperature over a period of 12 to 24 hours.

In all patients, blood pressure was continuously monitored using an arterial catheter, and target values for systolic and mean blood pressure were at least 90 and 75 mmHg, respectively. Hemodynamic monitoring was always performed using echocardiography.

### Data collection

Patient demographic data were collected for age, sex, medical history (hypertension, coronary heart disease and heart failure as assessed by New York Heart Association classification) and location of cardiac arrest (home, workplace or public place). Using information from the prehospital medical team, we also collected the duration of no-flow, the time to ROSC and the cumulative dose of epinephrine administered to the patient for initial resuscitation. In no case did patients receive norepinephrine or vasopressin. ECG results obtained immediately after ROSC were recorded and interpreted offline independently by a cardiologist to specifically determine the presence or absence of ST elevation and left bundle branch block (LBBB).

On ICU admission, the presence or absence of epinephrine infusion and the performance of coronary angiography with potential PCI was noted. Glasgow Coma Scale score, heart rate, core temperature and systolic, diastolic and mean arterial pressure were obtained. The ratio of partial pressure of arterial oxygen to the fraction of inspired oxygen (PaO_2_/FiO_2 _ratio), base deficit, lactate level and blood creatinine were also noted. Finally, the Simplified Acute Physiology Score II (SAPS II) was calculated [13].

### Patient outcomes

In-hospital mortality was reported. Surviving patients were routinely classified neurologically at the time of hospital discharge using the Cerebral Performance Categories (CPC) score [14] as follows: good cerebral performance (CPC 1), moderate cerebral disability (CPC 2), severe cerebral disability (CPC 3) or coma or vegetative state (CPC 4).

### Statistical analysis

Statistical analysis was performed by the Department of Biostatistics and Clinical Research Unit of the University Hospital Ambroise Paré (PA). Continuous variables were expressed as medians with 25th to 75th percentile ranges. Between-group comparisons were performed by means of the Wilcoxon rank-sum test for continuous variables and Pearson's χ^2 ^test for categorical variables. Age was considered as a continuous variable but also as a five-category ordinal one: age < 45 years (group 1), 45 to 54 years (group 2), 55 to 64 years (group 3), 65 to 74 years (group 4) and ≥75 years (group 5). All variables associated with survival with a *P *value less than 0.2 in a logistic regression equation were considered as candidates for the multivariate logistic model. For the assessment of continuous variables, we used the fractional polynomial method, an iterative estimation process that determines the best-fitting polynomial regression function, if any. Then the model was developed using a descending procedure. Last, interactions were tested, and goodness of fit was assessed by using the Hosmer-Lemeshow test. Statistical significance was reached if the two-tailed *P *value was < 0.05. Statistical analysis was performed using R statistical software (http://www.r-project.org/).

## Results

**Figure 1:**
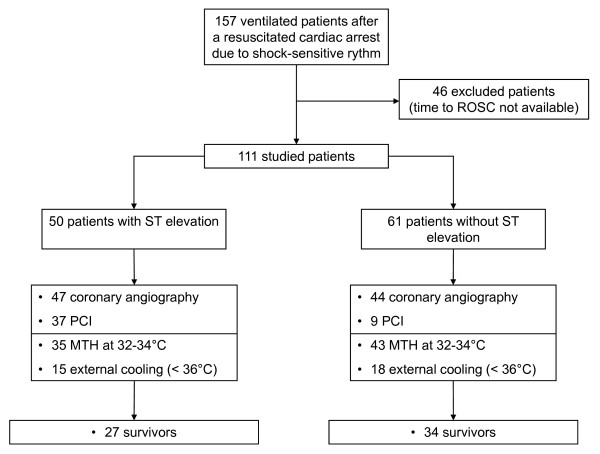
**Flow diagram of the study**. ROSC, return of spontaneous circulation; PCI, percutaneous coronary intervention; MTH, mild therapeutic hypothermia.

Among 157 eligible patients, 46 were not studied because the time to ROSC could not be precisely determined (mortality rate of excluded patients: 54%). A total of 111 patients were studied (87 men; mean age, 58 years (25th to 75th percentile range, 47 to 70 years)) (Figure 1). Twenty-two patients (20%) were in group 1, twenty-seven (24%) in group 2, twenty-two (20%) in group 3, twenty-three (21%) in group 4 and seventeen (15%) in group 5. The main characteristics of the overall population and according to age categories are reported in Tables 1 and 2. No-flow duration and time to ROSC were not different between the groups. Cardiac arrest was more likely to occur at home for older adults (group 5). Patients in group 1 (< 45 years old) were less likely to have ST-segment elevation or LBBB after resuscitation (Table 1).

**Table 1:** Prehospital characteristics of the study population^a^

Characteristics	Overall population	Group 1 (< 45 yr)	Group 2 (45 to 54 yr)	Group 3 (55 to 64 yr)	Group 4 (65 to 74 yr)	Group 5 (≥75 yr)
	(*N *= 111)	(*n *= 22)	(*n *= 27)	(*n *= 22)	(*n *= 23)	(*n *= 17)
Age, yr*	58 [47 to 70]	40 [36 to 43]	50 [47 to 52]	59 [58 to 61]	70 [67 to 72]	78 [76 to 83]
Males, *n *(%)	87 (78)	20 (91)	22 (81)	19 (86)	16 (70)	10 (59)
Medical history, *n *(%)						
Hypertension*	46 (41)	3 (14)	10 (37)	8 (36)	15 (65)	9 (53)
CHD*	27 (24)	2 (9)	4 (15)	3 (14)	10 (44)	8 (47)
NYHA classification III or IV*	29 (26)	1 (5)	5 (19)	5 (23)	12 (52)	6 (35)
Location of cardiac arrest*						
Home, *n *(%)	43 (39)	8 (36)	8 (30)	5 (24)	11 (48)	11 (65)
Workplace, *n *(%)	18 (16)	4 (18)	12 (44)	2 (10)	0	0
Public place, *n *(%)	50 (45)	10 (45)	7 (26)	15 (67)	12 (52)	6 (35)
No-flow duration, minutes	5 [1 to 10]	4 [1 to 8]	5 [1 to 10]	5 [1 to 10]	5 [1 to 10]	6 [1 to 10]
Time to ROSC, minutes	30 [16 to 45]	27 [14 to 44]	33 [19 to 48]	21 [19 to 50]	32 [16 to 45]	31 [19 to 41]
Epinephrine dose, mg	2 [0 to 8]	1.5 [0 to 7.5]	5 [0 to 10.5]	2 [0 to 5]	2 [0 to 4.5]	3 [0 to 6.25]
ECG, *n *(%)						
ST elevation	50 (45)	9 (41)	16 (59)	11 (50)	6 (26)	8 (47)
LBBB	10 (9)	0	2 (7)	3 (14)	4 (17)	1 (7)

^a^CHD: coronary heart disease; NYHA: New York Heart Association; ROSC: return of spontaneous circulation; epinephrine dose: cumulative dose of epinephrine required during initial resuscitation; ECG: electrocardiogram; LBBB: left bundle branch block. *P < 0.05 between-group comparisons. Numbers between brackets are percentages or 25th to 75th percentiles.

**Table 2:** Characteristics of study population on ICU admission and outcome^a^

Characteristics	Overall population	Group 1 (< 45 yr)	Group 2 (45-54 yr)	Group 3 (55 to 64 yr)	Group 4 (65 to 74 yr)	Group 5 (≥75 yr)
	(*N *= 111)	(*n *= 22)	(*n *= 27)	(*n *= 22)	(*n *= 23)	(*n *= 17)
SAP, mmHg	124 [107 to 146]	117 [107 to 140]	124 [113 to 138]	137 [121 to 158]	124 [106 to 142]	120 [104 to 146]
Heart rate, beats/min	91 [77 to 107]	94 [80 to 112]	92 [83 to 108]	93 [80 to 110]	85 [74 to 93]	82 [61 to 101]
Glasgow Coma Scale score	3 [3 to 3]	3 [3 to 3]	3 [3 to 3]	3 [3 to 3]	3 [3 to 3]	3 [3 to 3]
Base deficit, mmol/L	6.4 [3 to 10.4]	5.8 [2.6 to 11.4]	6.9 [4.4 to 11.4]	5.5 [3 to 10.9]	6.9 [1.4 to 10.4]	6.6 [3.3 to 9.2]
PaO_2_/FiO_2 _ratio, mmHg	253 [174 to 337]	234 [192 to 319]	294 [190 to 348]	205 [141 to 283]	248 [153 to 327]	255 [208 to 325]
Catecholamine infusion	62 (56)	14 (64)	14 (52)	12 (55)	14 (61)	8 (47)
Creatinine, μmol/L	111 [86 to 131]	98 [83 to 124]	114 [83 to 144]	112 [87 to 122]	126 [101 to 160]	109 [95 to 124]
SAPS II*	60 [52 to 68]	53 [48 to 62]	56 [51 to 66]	57 [48 to 61]	62 [59 to 73]	72 [63 to 79]
Coronary angiography*	91 (82)	21 (95)	25 (93)	20 (91)	16 (70)	9 (53)
PCI in patients with coronary angiography	46 (51)	7	14	12	9	4
MTH 32°C to 34°C*	78 (70)	19 (86)	21 (78)	17 (77)	13 (57)	8 (47)
In-hospital survival**	60 (54)	14 (64)	14 (52)	14 (64)	14 (61)	4 (24)

^a^SAP: systolic arterial pressure; PaO_2_/FiO_2 _ratio, ratio of partial pressure of arterial oxygen to the fraction of inspired oxygen; SAPS II: Simplified Acute Physiology Score II; PCI: percutaneous coronary intervention; MTH: mild therapeutic hypothermia. *P < 0.05 between-group comparisons. **P = 0.06 between-group comparisons. Numbers between brackets are percentages or 25th to 75th percentiles.

On ICU admission, the main characteristics of the study population were similar between groups, with the exception of SAPS II, which was higher in group 5 because of the impact of age (Table 2). MTH was performed in 96 patients (86%). In the remaining patients, MTH was not performed because of severe hemodynamic instability or moribund status. Target temperature (32°C to 34°C) was reached in 78 patients (81%) (Table 2). In the remaining 18 patients (19%), core temperature was always maintained below 36°C. Coronary angiography was not performed in 20 patients (18%) because of hemodynamic instability, with six of them considered as moribund by the attending physician (four of them were in group 5). Among the remaining 91 patients, 40% had one-vessel disease, 34% had two-vessel disease and 26% had three-vessel disease. Of these patients, 46 (51%) underwent PCI for acute coronary occlusion (Table 2). PCI was successful in 94% of the cases and involved the left anterior descending artery (51%), the circumflex (11%), the right coronary artery (25%) or cardiac bypass grafts (13%). With a single exception, all patients in group 1 (< 45 years old) underwent emergent angiography, which was associated with a PCI in seven of them (33%). In group 5 (≥75 years old), only nine patients (53%) underwent coronary angiography, but seven (44%) had a PCI. In-hospital survival was lower in group 5 (≥75 years), but without reaching statistical significance (Table 2).

Fifty patients (45%) exhibited ST-segment elevation on the ECG recorded immediately after ROSC. Forty-seven (94%) of them underwent coronary angiography, and 37 patients (74%) had a PCI. Forty-five patients (73%) with non-ST elevation underwent emergent coronary angiography, and nine patients (15%) benefited from a PCI. Among patients with or without ST-segment elevation, no statistically significant difference was found for age, time to ROSC, SAPS II, MTH or survival (data not shown).

**Figure 2:**
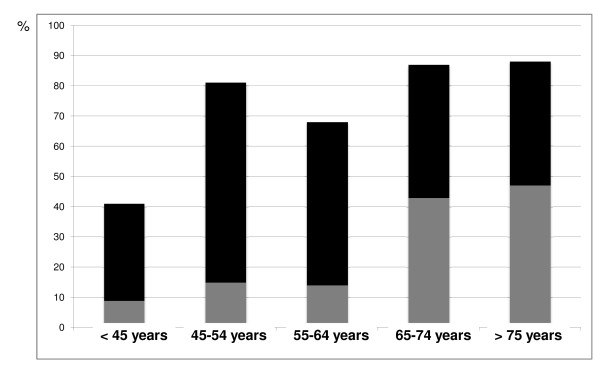
**Incidence of documented coronary heart disease according to age and before (gray) and after (black) coronary angiography**.

Figure 2 depicts the incidence of known coronary heart disease before and after coronary angiography according to age. Most patients (73%) had coronary heart disease, although the incidence in group 1 (< 45 years) was significantly lower than that in other groups (41% versus 81%; *P *= 0.01). Angiography revealed previously unknown coronary heart disease in 54 patients (49%). This diagnosis was more frequently unsuspected in groups 1, 2 and 3 than in groups 4 and 5 (Figure 2).

**Figure 3:**
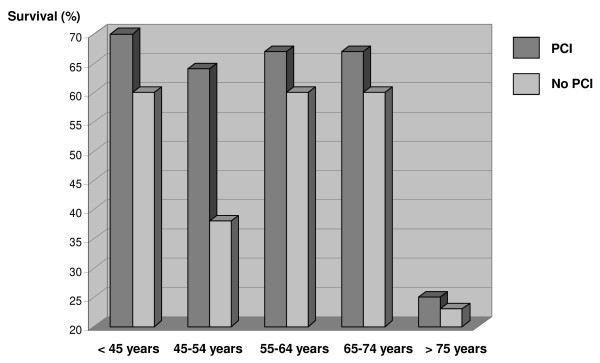
**Survival according to whether a percutaneous coronary intervention (PCI) was performed in the different study groups**.

Overall in-hospital survival was 54%. Of the surviving patients, six (10%) were classified as CPC 3 or 4 and fifty-four (90%) as CPC 1 or 2. Table 3 reports intergroup differences between surviving and deceased patients. Age, time to ROSC, SAPS II, coronary angiography, PCI, MTH, cumulative epinephrine dose during initial resuscitation, serum creatinine, base deficit and PaO_2_/FiO_2 _ratio were entered into a multivariate logistic regression model. Time to ROSC was significantly associated with mortality and PCI with survival with odds ratios (ORs) of 1.05 (25th to 75th percentile range, 1.03 to 1.08; *P *< 0.001) and 0.30 (25th to 75th percentile range, 0.11 to 0.79; *P *= 0.01), respectively. No transformation of the two raw variables reached significance. Age did not reach significance (*P *= 0.17) with an OR of 1.022 (25th to 75th percentile range, 0.99 to 1.05), despite a trend toward a decrease in survival in patients ≥75 years of age (Table 2). Figure 3 depicts survival rate according to PCI.

**Table 3:** Between-group differences in surviving and deceased patients^a^

Characteristics	Surviving (*n *= 60)	Deceased (*n *= 51)	*P *value
Males, *n *(%)	47 (78)	40 (78)	0.83
Age, yr	56 [46 to 67]	61 [50 to 74]	0.07
SAPS II	56 [48 to 64]	63 [58 to 74]	0.0005
Duration of no-flow, minutes	4 [1 to 10]	7 [1 to 10]	0.1
Time to ROSC, minutes	20 [15 to 35]	40 [27 to 56]	0.00005
Epinephrine dose, mg	0 [0 to 4]	5 [1 to 10]	0.005
Location of cardiac arrest			0.44
Home	21 (36)	22 (44)	
Workplace	12 (20)	6 (12)	
Public place	27 (45)	23 (45)	
Coronary angiography	54 (90)	37 (73)	0.03
PCI	29 (48)	17 (33)	0.15
MTH	46 (77)	32 (63)	0.16
Glasgow Coma Scale score	3 [3 to 4]	3 [3 to 3]	0.07
SAP, mmHg	129 [114 to 148]	121 [105 to 146]	0.21
Heart rate, beats/min	91 [78 to 104]	88 [74 to 108]	0.9
PaO_2_/FiO_2 _ratio, mmHg	258 [194 to 340]	241 [127 to 328]	0.13
Creatinine, μmol/L	102 [83 to 120]	118 [98 to 147]	0.02
Base deficit, mmol/L	4.9 [2.1 to 9]	7.2 [3.9 to 11.6]	0.18

^a^SAPS II: Simplified Acute Physiology Score II; ROSC: return of spontaneous circulation; epinephrine dose: cumulative dose of epinephrine required during initial resuscitation; PCI: percutaneous coronary intervention; MTH: mild therapeutic hypothermia; SAP: systolic arterial pressure; PaO_2_/FiO_2 _ratio, ratio of partial pressure of arterial oxygen to the fraction of inspired oxygen. Numbers between brackets are percentages or 25th to 75th percentiles.

## Discussion

In the present study, routine coronary angiography after OHCA related to VF, regardless of ECG pattern (that is, ST elevation or not), allowed us to detect and to treat acute coronary occlusion in 46 (41%) of the 111 studied patients. Importantly, PCI was independently associated with in-hospital survival in our patients with stabilized hemodynamics.

The present overall survival rate of 54% is markedly higher than that reported in previous series where neither emergent coronary angiography nor MTH were used. Cobb *et al*. [15] and Greene *et al*. [16] reported survival rates of 29% and 26%, respectively, in patients with out-of-hospital resuscitated cardiac arrest. In 1997, Spaulding *et al*. [8] suggested that routine coronary angiography, associated with PCI when necessary, may improve patient prognosis, with a 38% survival rate in a population between 30 and 75 years of age. However, MTH was not yet used at the time as a standard of care. In the Spaulding *et al*. study, 60 (71%) of 84 patients had significant coronary heart disease on the basis of angiography and 61% of them underwent a PCI [8]. In keeping with these results, 91 patients in the present series underwent emergent coronary angiography, and 51% of them benefited from associated PCI for the presence of underlying acute coronary occlusion. Recently, two randomized, controlled studies demonstrated that MTH increases survival rate with a good neurological outcome in patients who have sustained cardiac arrest secondary to VF [4,5]. Accordingly, MTH and coronary angiography are now recommended in adult patients under 75 years old following cardiac arrest related to VF in the presence of a suspected acute coronary syndrome with ST elevation [2]. Sunde *et al*. [7] reported that this therapeutic strategy significantly increased survival from 26% in a control group to 56% in a group of patients who received a standardized treatment. In the control group, however (the one without MTH and angiography), only 48% of patients were < 70 years of age, whereas 71% in the intervention group with MTH and angiography were < 70 years of age, rendering data interpretation difficult.

In keeping with previous studies [8,17], we have shown that PCI is strongly and independently associated with survival in patients with stable hemodynamics. In addition, routine coronary angiography allowed us to diagnose previously unknown significant coronary heart disease in 49% of our patients, especially those < 65 years old (groups 1, 2 and 3), regardless of the presence of ST elevation. Dumas *et al*. [17] recently reported similar results in a large series of patients with OHCA related to shock-sensitive (68%) and unshockable (32%) rhythms. In patients with no obvious extracardiac etiology and no ST elevation visualized on ECGs, routine coronary angiography disclosed coronary heart disease in 58% of patients and PCI was performed in 26% of them. Successful PCI also appeared to be protective in this series [17]. Similarly, Lellouche *et al*. [18] reported, in a large series of patients resuscitated after cardiac arrest who systematically underwent a coronary angiography, that myocardial infarction was the leading cause (54%) and that the sensitivity and specificity of ST elevation to detect the presence of an underlying acute coronary lesion were 50% and 88%, respectively. Taken together, these data strongly support the performance of a routine coronary angiography in the clinical setting of resuscitated patients who are treated with MTH after OHCA secondary to VF. Nevertheless, discrepant results have previously been reported in similar clinical settings. In a series of 186 patients who underwent immediate PCI after successful resuscitation for cardiac arrest complicating acute myocardial infarction, Garot *et al*. [9] showed that PCI was not associated with survival. Similarly, Anyfantakis *et al*. [10] reported that the use of PCI was not an independent correlate of survival in 72 patients who underwent immediate coronary angiography after being resuscitated from cardiac arrest. Importantly, coronary angiography was systematically performed in these studies, whereas this procedure was carried out only in patients with stable hemodynamics in the current series. In addition, the need for epinephrine infusion during coronary angiography was strongly associated with death in the previous work reported by Anyfantakis *et al*. [10].

There are conflicting data regarding the impact of age on the prognosis of patients who have sustained OHCA related to VF [19,20]. Most studies which have assessed this potential relationship have been performed before the initiation of MTH and coronary angiography [21] or involved only patients < 75 years of age [8]. In a recent study, age was not a prognostic factor [22]. However, studied patients were < 75 years old, and VF was the initial cardiac rhythm in only 42% of them [22]. In contrast, studies performed prior to the early initiation of MTH and PCI strongly suggested a relationship between patient age and postresuscitation mortality [23,24]. In patients who sustained OHCA or in-hospital cardiac arrest, Nolan *et al*. [25] recently reported that the OR for death reached 1.16 for each five-year increase above 31 years of age. Nevertheless, only 14% of patients in this population had documented VF [25]. Dumas *et al*. [17] reported that age > 59 years was independently associated with mortality, but > 30% of patients had an unshockable rhythm. Although the survival rate was as low as 24% in patients ≥75 years old compared to 60% in other groups of the current series, age was not independently associated with mortality when considered as a continuous variable or as a five-category ordinal one. This result may be related to the fairly homogeneous practice of coronary angiography and potentially associated PCI regardless of age, since it was performed in 70% to 95% of patients < 75 years old. Noticeably, coronary angiography was performed in only 53% of patients ≥75 years old, despite the practice of this invasive procedure as a standard of care in participating ICUs. Namely, six of nine older adult patients who did not benefit from emergent coronary angiography were considered moribund or too hemodynamically unstable by the attending physician. When considering the high mortality rate (76%) observed in this group of patients, the clinical relevance of routine coronary angiography after a resuscitated cardiac arrest secondary to VF in patients ≥75 years old is questionable.

The main limitation of our study is related to its observational design, precluding the constitution of a control group. Since time to ROSC was not determined in 46 cases, we could not include this subset of patients in the analysis. Nevertheless, the mortality rate in excluded patients (54%) was similar to that of the study population (46%). The lack of power of the study due to the enrollment of only 17 patients in the older adult population (≥75 years) does not allow us to offer definite recommendations on the clinical relevance of routine coronary angiography in this group. Emergency coronary angiography was not performed in hemodynamically unstable patients. With the exception of moribund patients, hemodynamics may have been restored more efficiently with the use of PCI in severely hypotensive patients. Accordingly, the results of our multivariate analysis hold true only in patients with stabilized hemodynamics. Finally, the insertion of an intraaortic balloon pump during PCI was not recorded, and its potential influence on outcome was therefore not addressed.

## Conclusions

We suggest that routine coronary angiography with potentially associated PCI may alter the prognosis of OHCA related to VF in resuscitated patients with stable hemodynamics who are treated with MTH. Whether this strategy is clinically relevant in patients ≥75 years old remains to be determined by further studies.

## Key messages

• Routine coronary angiography leading to PCI was independently related to survival in our cohort of resuscitated patients with stabilized hemodynamics who underwent MTH after an OHCA due to VF.

• Whether this therapeutic strategy favorably alters the prognosis of older adult patients after an OHCA secondary to a shock-sensitive rhythm remains to be determined.

## Abbreviations

ECG: electrocardiogram; ICU: intensive care unit; MTH: mild therapeutic hypothermia; PCI: percutaneous coronary intervention; ROSC: return of spontaneous circulation; SAPS II: Simplified Acute Physiology Score II; VF: ventricular fibrillation.

## Competing interests

The authors declare that they have no competing interests.

## Authors' contributions

PC, KB, CC, FT, SC, REM, CL and NP contributed to the acquisition of data. PA performed data analysis. PV, OD and AVB participated in the conception of the study, the interpretation of data and the elaboration of the draft and of revisions of the manuscript. All authors read and approved the final manuscript.
